# Perinatal and Antibiotic Exposures and the Risk of Developing Childhood-Onset Inflammatory Bowel Disease: A Nested Case-Control Study Based on a Population-Based Birth Cohort

**DOI:** 10.3390/ijerph17072409

**Published:** 2020-04-02

**Authors:** Cristina Canova, Jonas F Ludvigsson, Riccardo Di Domenicantonio, Loris Zanier, Claudio Barbiellini Amidei, Fabiana Zingone

**Affiliations:** 1 Unit of Biostatistics, Epidemiology and Public Health, Department of Cardio-Thoraco-Vascular Sciences and Public Health, University of Padua, 35131 Padua, Italy; claudioamidei@gmail.com; 2 Department Medical Epidemiology and Biostatistics, Karolinska Institutet, SE-171 77 Stockholm, Sweden; jonasludvigsson@yahoo.com; 3 Department of Pediatrics, Örebro University Hospital, Örebro University, 701 81 Örebro, Sweden; 4 Division of Epidemiology and Public Health, School of Medicine, University of Nottingham, Nottingham NG7 2UH, UK; 5 Department of Medicine, Columbia University College of Physicians and Surgeons, New York, NY 10032, USA; 6 Department of Epidemiology, Lazio Regional Health Service, ASL Roma 1, 00147 Rome, Italy; r.didomenicantonio@deplazio.it; 7 Epidemiological Service, Health Directorate, 35131 Udine, Italy; loris.zanier110@gmail.com; 8 Department of Surgery, Oncology and Gastroenterology, Gastroenterology Section, University Hospital of Padua, 33100 Padua, Italy; fabiana.zingone@unipd.it

**Keywords:** pediatric IBD, VEO-IBD, Crohn disease, ulcerative colitis, birth cohort study, epidemiology, real-world data, record linkage, perinatal and postnatal exposure, pharmacoepidemiology

## Abstract

The role of early-life environmental exposures on Inflammatory Bowel Disease (IBD) onset remains unclear. We aimed to quantify the impact of perinatal conditions and antibiotic use in the first 6 and 12 months of life, on the risk of childhood-onset IBD, in a birth cohort of the region Friuli-Venezia Giulia (Italy). A nested case-control design on a longitudinal cohort of 213,515 newborns was adopted. Conditional binomial regression models were used to estimate Odds Ratios (OR) with 95% confidence intervals (CI) for all analyzed risk factors. We identified 164 individuals with IBD onset before the age of 18 years and 1640 controls. None of the considered perinatal conditions were associated with IBD. Analyses on antibiotic exposure were based on 70 cases and 700 controls. Risks were significantly higher for children with ≥4 antibiotic prescriptions in the first 6 and 12 months of life (OR = 6.34; 95%CI 1.68–24.02 and OR = 2.91; 95%CI 1.31–6.45, respectively). This association was present only among patients with Crohn’s disease and those with earlier IBD onset. We found that perinatal characteristics were not associated to IBD, while the frequent use of antibiotics during the first year of life was associated to an increased risk of developing subsequent childhood-onset IBD.

## 1. Introduction

Inflammatory bowel disease (IBD), comprising Crohn’s disease and ulcerative colitis, is characterized by chronic inflammation of the gastrointestinal tract that can occur at any age, with a peak of incidence between 15 and 29 years, but with a considerable number of cases developing in childhood [1,2]. Differences in the clinical progression of childhood-onset IBD have been observed according to age at onset and have been categorized in Pediatric IBD (P-IBD) between 10–17 years, Early-Onset IBD (EO-IBD) less than 10 years and Very Early-Onset IBD (VEO-IBD) less than 6 years [3,4]. The incidence of IBD in the pediatric population is increasing, especially in Western countries, with a recent study reporting a stable disease incidence before 6 years of age [5], but an increase between 6 and 16 years [6]. 

IBD is multifactorial resulting from a confluence of genetic, microbial and environmental factors. Smoking [7], oral contraceptives [8], dietary fat intake [9] and appendectomy [10,11] have all been identified as potential later-life environmental risk factors in IBD. Amongst early-life environmental exposures, no risk factors have been firmly established, while breastfeeding seems to have a protective role in preventing childhood onset IBD [12,13] and cesarean delivery [14,15] and birth weight [16] have shown no association. According to the “hygiene hypothesis”, individuals raised in an overly sanitized environment have a higher risk of developing IBD, even if existing studies are discordant. Several works have explored the role of antibiotic use on IBD onset due to their possible effect on the composition of infant gut microbiome in both an immediate [17,18] and sustained manner [19,20]. Most papers focused on adult exposure to antibiotics, while few have considered exposures in the first year of life [21,22,23,24]. Even fewer papers have distinguished between age at IBD onset (EO-IBD, P-IBD and VEO-IBD) [23].

Our study aims to quantify the impact of perinatal conditions and antibiotic exposure in the first 6 and 12 months of life, on the risk of developing EO-IBD (<10 years), P-IBD (10–17 years), Crohn’s disease and ulcerative colitis, in a cohort of newborns in the Region of Friuli-Venezia Giulia (Italy). 

## 2. Methods 

The study was conducted in Friuli-Venezia Giulia, in North-East Italy, which has about 1.2 million inhabitants with about 10,000 births per year. A matched case-control design, nested in a cohort linked to administrative data, was adopted. The original cohort included 213,515 individuals (excluding abortions, stillbirths and deaths in the first year of life) born between 1989 and 2012, resident in the region of Friuli-Venezia Giulia [25]. Only individuals with an IBD diagnosis before the age of 18 years were included. 

Data on the population derives from the regional medical birth register [26], where hospital and home deliveries are recorded for the entire population. This register contains data on the socioeconomic status of the parents, information on pregnancy, labor and delivery, as well as data on the newborn. 

The National Health Service (NHS) in Italy is organized at a regional level and provides universal, free-of-charge coverage for general practitioner and hospital services, to all Italian and European Union citizens resident in the country, regardless of income. Specific drugs for chronic disease treatment as well as specialist visits are guaranteed free of charge by the NHS through disease-specific healthcare co-payment exemptions, provided that the clinical condition is diagnosed and certified by an NHS specialists. In Friuli-Venezia Giulia, a regional integrated healthcare system developed in the 1980s collects and pools automatically data on healthcare services provided by the NHS. Each individual is identified by means of a unique, anonymous, regional code that has been described in a previous paper [25].

In this study, we used healthcare data from the following databases: population registry; mortality records; hospital discharge records with information collected during episodes of inpatient care, including day hospital records, that occurred within or outside of the region, with up to 6 diagnoses coded according to the International Classification of Diseases, Ninth Revision, Clinical Modification (ICD-9-CM); healthcare co-payment exemptions, based on a national coding system; drug prescription records, coded according to the Anatomical Therapeutic Chemical (ATC) Classification System (available from 1995 in the region of Friuli-Venezia Giulia).

This retrospective study was approved by the Institutional Review Board of the University of Padua (Italy). No informed consent and no Ethics Committee approval were required because this record linkage study was based on computerized databases of medical records and all the data was de-identified prior to analysis.

### 2.1. IBD Case Identification

Cases affected by IBD before the age of 18 years were defined as having at least one of the following: a hospital discharge record with an ICD-9-CM code 555 (regional enteritis) or 556 (ulcerative colitis), but not 556.0 (chronic ulcerative enterocolitis); 556.1 (chronic ulcerative ileocolitis); 556.4 (colonic pseudopolyposis); 556.8 (other ulcerative colitis), in any of the up to 6 possible diagnoses recorded; an exemption for healthcare co-payment with code 009.555 (for both Crohn’s disease and ulcerative colitis) according to the Italian national coding system. This case-identification algorithm is based on a previously validated one that reported a sensitivity of 82.2% for hospital discharge records and healthcare co-payment exemptions combined and a sensitivity of 75.4% exclusively for hospital discharge records [27]. IBD onset age was defined as the earliest date identifiable among the used sources of information (hospital admission or healthcare co-payment exemption).

Individuals affected by IBD were divided according to age at IBD onset in two groups of Early Onset IBD (EO-IBD) with <10 years (Paris classification A1a) and Pediatric IBD (P-IBD) between 10 and 17 years (Paris classification A1b).

### 2.2. Perinatal Conditions and Antibiotic Exposures 

Among the medical birth register data, we considered the following variables as possible risk factors: season of birth (May–September vs. October–April); having older siblings (no vs. yes); multiple births (≥2 vs. 1); birth weight (<2500 g vs. ≥2500 g); gestational age (≤35 vs. ≥36 weeks); Apgar scores at 1 minute (<7 vs. ≥7); maternal age (<25, 30–34, 35–39, ≥40 vs 25–29 years); and mother’s formal education at the moment of birth (university, high school vs. primary/middle school). Data on type of delivery (spontaneous, planned cesarean, unplanned cesarean or other) was only available from 2002 onwards and it was not considered for these analyses.

Data on antibiotic use (ATC code J01*) in the first 6 and 12 months of life was identified from the drug prescription database. This archive, available since 1995, provides a complete coverage of all antibiotics dispensed by pharmacies under medical prescription. All antibiotic prescriptions are fully reimbursed by the NHS. Drug prescriptions records have been previously used in other papers that analyzed exposure to antibiotics in the first year of life [28].

### 2.3. Data Analysis

A nested matched case-control study design was adopted. By adopting a SAS (Statistical Analysis System) macro through an iterative process, all IBD cases were identified from the cohort. In the same population, for each case, all possible controls were identified among newborns recorded in the medical birth register (alive at 1 year of age, after merging with mortality records), alive and resident in the region on the date of IBD diagnosis (index date) and matched for sex and year of birth. Ten controls were randomly selected for each case. Each control was assigned an index date, that corresponded to the date of IBD diagnosis of the matched case. 

Conditional binomial regression models were used to estimate Odds Ratios (OR) with 95% confidence intervals (CI) for each possible perinatal condition and antibiotic exposure associated with IBD diagnosis, among the matched case-control pairs. This way, age and sex confounding was always controlled by the study design. Models included as possible confounding factors all the individual perinatal characteristics (season of birth; having older siblings; multiple births; birth; gestational age; Apgar scores at 1 minute; maternal age; and mother’s formal education at the moment of birth) identified in the medical birth register. 

**Figure 1:**
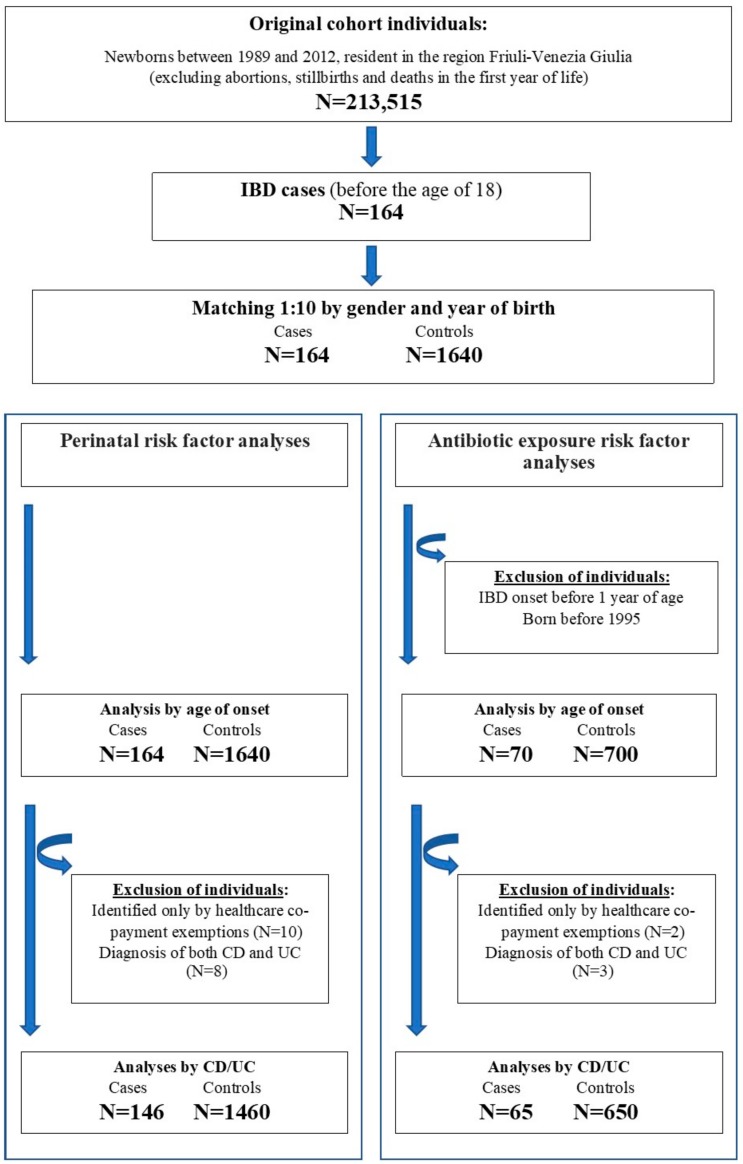
Flow chart of patients with Inflammatory Bowel Disease (IBD), Crohn’s Disease (CD) and Ulcerative Colitis (UC) in the study.

We conducted two main analyses, both stratified by age at onset (<10 years EO-IBD and 10–17 years P-IBD) and type of IBD (Crohn’s disease and ulcerative colitis), to estimate the association between perinatal conditions and antibiotic exposures with the development of IBD (see Figure 1). Analyses stratified by Crohn’s disease and ulcerative colitis were restricted to individuals identified by means of hospital discharge records, since healthcare co-payment exemptions had the same code and did not allow us to distinguish between the two conditions. Individuals with both a diagnosis of Crohn’s disease and ulcerative colitis in hospital discharge records were also excluded from the analyses (Figure 1).

For analyses related to antibiotic exposure, we excluded all individuals with IBD onset (index date in control individuals) before 1 year of age, or born before 1995 due to the unavailability of data on drug prescriptions before that year. In order to control for another possible risk factor and to reduce possible confounding by indication, we fitted another multivariate model that included gastrointestinal infections (ICD-9-CM codes 001-009 in any of 6 possible diagnoses) that required hospital admission during the first 12 months of life. To assess potential causality, the dose–response association between antibiotic consumption and IBD onset was examined considering the number of prescriptions as a categorical (0, 1, 2–3 and 4+) variable. A first sensitivity analysis only restricted to antibiotic exposure, was performed on individuals with Very Early Onset-IBD—(VEO-IBD), with an IBD diagnosis between 1 to 5 years of age. A further sensitivity analysis was performed on a subgroup of subjects with VEO-IBD considering only IBD onset between 2 to 5 years of age, to reduce the risk of a possible confounding by indication of antibiotics. Statistical analyses were conducted with SAS software version 9.4 (SAS Institute, Cary, NC, USA).

## 3. Results 

The cohort consists of 213,515 individuals, born between 1989 and 2012 and resident in Friuli-Venezia Giulia (Italy). Through hospital discharge records or healthcare co-payment exemptions, we identified 164 individuals with IBD onset before the age of 18 years and 1640 references, matched by sex and year of birth. The exclusive contribution of hospital discharge records was 40.2%, whereas 53.7% were identified by both hospital discharge records and healthcare co-payment exemptions and only 6.1% were identified exclusively by means of healthcare co-payment exemptions. Through hospital discharge records, it was possible to distinguish between Crohn’s disease (83 cases 53.9%) and ulcerative colitis (63 cases 40.9%) (Figure 1). 

### 3.1. Perinatal Exposure

Analyses on perinatal exposures were based on all individuals identified as affected by IBD (n = 164), as described in Table 1. In this population, the mean age among cases was 10.2 years (SD 5.2) and the same characteristics applied to controls matched by age and gender. None of the studied characteristics were associated with the development of any type of IBD (Table 2) nor with any of the IBD subgroups that have been analyzed (Table S1 and Table S2).

### 3.2. Antibiotic Exposure

The analyses on antibiotic exposure in the first year of life was based on 70 cases and 700 controls, all born after 1995 (Table 1 and Table 3). The mean age of IBD cases was 8.8 years (SD 4.7). Overall, in the first 12 months of life, 749 antibiotics had been prescribed (125 among cases and 624 among controls) as shown in Table S3. The most commonly prescribed antibiotics were penicillins (53.3%, particularly amoxicillin), followed by cephalosporins (25.8%) and macrolides (18.3%). Only one prescription of quinolone and no prescriptions for metronidazole have been observed. 

At least one antibiotic had been prescribed to 47.1% of cases and 45.7% controls (adj2 OR = 1.08; 95%CI 0.64-1.80, *p*-value = 0.78), as shown in Table 3. Risk estimates were significantly higher among individuals with ≥4 antibiotic prescriptions across all subgroups and after adjusting for gastrointestinal infections in the first 12 months of life, risks remained elevated (adj3 OR = 2.91; 95%CI 1.31–6.45, *p*-value = 0.003). The 11 individuals with ≥4 drug prescriptions in the first 12 months of life had received 92 drug prescriptions of which 50% were amoxicillin (with or without beta-lactamase inhibitor), 23% cefaclor and 11% gentamicin.

Stratified analyses showed especially high risks in children with EO-IBD (Table 4). Exposure to antibiotics in the first 6 months of life was generally associated with higher risks of childhood onset IBD, in all analyzed subgroups (Table 3, Table 4 and Table 5). 

The sub-analyses on the type of IBD lead to identify 38 cases (55.9%) affected by Crohn’s disease and 27 cases (39.7%) affected by ulcerative colitis, as shown in Table 5. Significantly increased risks following antibiotic exposure were present only among individuals with Crohn’s disease and especially high for subjects exposed in the first 6 months of life (adj2 OR = 2.61; 95%CI 1.17–5.81, *p*-value = 0.02) as reported in Table 5. Risks associated to the development of Crohn’s disease were especially elevated for individuals exposed to ≥4 antibiotic prescriptions both in the first 6 and in the first 12 months of life (respectively adj2 OR = 13.02; 95%CI 2.47–68.47, *p*-value = 0.01 and adj2 OR = 4.86; 95%CI 1.70–13.90, *p*-value = 0.005) as shown in Table 5.

A sensitivity analysis for individuals affected by VEO-IBD (n = 19) showed markedly increased risks for antibiotic exposure in the first 6 months of life (adj2 OR = 4.56; 95%CI 1.48–14.02, *p*-value = 0.01) and in the first 12 months of life (adj2 OR = 4.54; 95%CI 1.47–14.02, *p*-value = 0.01), especially after ≥4 antibiotic prescriptions, although risks were based on a very small number of cases (Table S4). A further sensitivity analysis has been performed, by excluding cases diagnosed with IBD before the age of 2 years. These analyses also showed elevated risks, but failed to attain statistical significance, except for individuals with ≥4 antibiotic prescriptions (data not shown).

## 4. Discussion

### 4.1. Main Results

In this cohort study, we found a strong association between the increasing number of antibiotic prescriptions in the first year of life and childhood onset IBD. This association seemed to be present only among patients with Crohn’s disease and risks were increasingly higher for earlier diagnoses (VEO-IBD > EO-IBD > P-IBD). Risks were generally higher following exposures in the first 6 months of life. Most IBD patients had taken penicillins followed by cephalosporins and macrolides, while only one patient had been prescribed a quinolinic antibiotic and no patient had received metronidazole. This association remained significant even after adjusting for gastrointestinal infections in the first year and after excluding cases with IBD onset before 2 years of age. We believe this analysis strongly reduced the possibility of reverse causation, since some antibiotics, in particular metronidazole and quinolones, might be used for symptoms correlated to IBD, that are misdiagnosed as gastrointestinal infections. On the other hand, our study did not find an association between childhood onset IBD and any of the other analyzed perinatal conditions, moreover such factors do not seem to play a role on the association between antibiotic utilization and IBD. 

### 4.2. Previous Literature

The study of possible early risk factors for IBD onset represents, nowadays, a topic of relevant interest. According to a systematic review by Cholapranee et al., there is an inverse association between bedroom sharing, number of siblings, and the risk of IBD [29]. In support of the “hygiene hypothesis” a recent population-based study [30] on 825 IBD cases and 5999 matched controls found that being in the highest socioeconomic quintile at birth increased risk of developing IBD at some point in life. The same study found that early-life infections and the presence of a mother affected by IBD increased the risk of developing IBD later in life. In accordance with our study, no association of IBD was found with early-life conditions such as birth weight, Apgar at birth and gestational age. Conversely, another recent work [31] did not confirm the role of the “hygiene hypothesis” in IBD. We also did not find any association between childhood onset IBD and season of birth, in contrast with previous studies [32,33].

Previous literature has mainly focused on the risk of antibiotic use and the development of IBD, but only few papers have specifically analyzed the association between antibiotic exposure in the first year of life with the subsequent risk of IBD onset in different periods of infancy and adolescence. 

Shaw et al. found that 58% of IBD cases (mean age at diagnosis 8.4 years), mainly affected by Crohn’s disease, had ≥1 antibiotic dispensation in their first year of life, as opposed to 39% of controls [21]. Two years later, Kronman et al. observed that antibiotic exposure before 1 year of age was associated with a hazard ratio of developing IBD of 5.5, compared with hazard ratios of 2.6 and 1.6 in children who received antibiotics before the age of 5 and 15, respectively. They also described a dose-dependent effect with respect to the number of antibiotics taken [22]. In the same period two register-based studies described an increased risk of Crohn’s disease among children following antibiotic exposure earlier in life [34,35]. Similarly, antibiotic use years prior to diagnosis has been associated with IBD onset and particularly Crohn’s disease in adults [24,36,37,38]. In 2014, Ungaro et al performed a metanalysis including more than 7000 IBD patients showing an association between antibiotic exposure and the development of Crohn’s disease, but not ulcerative colitis and the association was especially marked in children. All antibiotics were associated with IBD, except for penicillins. A stronger association was found with the use of metronidazole or fluoroquinolones compared to all other antibiotics [39]. However, this meta-analysis included articles with different study designs and study populations, in particular considering patients exposed to antibiotics at any age before IBD diagnosis. This is especially relevant because the type of antibiotic used and its indication can change according to the age. Among neonates, the early development of the microbiota has been observed to change between subjects treated with antibiotics and those not treated [40,41]. More recently, Troelsen et al. found only a slight increase in the risk of developing Crohn’s disease among subjects exposed to quinolones and metronidazole at any time in life, compared to subjects who were never exposed to these drugs. When the authors restricted the analyses to individuals followed from birth, the use of any antibiotic before the age of 5 was associated to IBD onset, even if not statistically significant (OR = 2.20; 95% CI 0.75–6.43) [24]. In 2019 Örtqvist et al. [23] conducted a study on a cohort of 827,239 children born in Sweden between 2006 and 2013 focusing on the risk of IBD onset before 6 years of age (VEO-IBD). The authors found an HR of 1.49 (95% CI 0.69 to 3.22) looking at the association between individuals who had been exposed to systemic antibiotics during the first year of life and the risk of IBD onset from 2 years of age. This HR was similar to the one found in our study when considering only one prescription, however, as oppose to our findings, no dose-response relationship was observed (although this analysis did not restrict exposures to the first year of life). Murine studies have also shown that a physiological development of the microbial flora during the neonatal period is important to assure a normal presence of natural killer T cells in the gut that provide greater protection against colitis [42].

### 4.3. Strengths and Limitations 

The main strength of our study is the population-based design with a long follow-up where all data was prospectively collected eliminating the risk of recall bias and we used a validated IBD definition to identify all cases. We observed a higher prevalence in males than in females at all ages, for both Crohn’s disease and ulcerative colitis. This is only partially in line with a recent pooled analysis on IBD prevalence in Western countries that showed more male children affected by Crohn’s disease among those aged 0–14 years, while there were slightly more female subjects affected by ulcerative colitis among those aged 0–9 [43]. Moreover, we looked at early antibiotic use (first 6 months of life), when antibiotics can particularly influence the microbiota [40]. In addition, the effect of reverse causation in our results has been reduced by adjusting our analyses for gastrointestinal infections that occurred in the first 12 months of life, and by sub-analyses exploring the risk of IBD after 2 years of age, therefore, further away from the exposure of interest. Another important strength point of our study is the evaluation of the effect of antibiotic exposure according to the different age at onset (VEO-IBD, EO-IBD and P-IBD). As oppose to previous evidences, we can also conclude that our results are attributable to the use of the most common classes of antibiotics prescribed during the first year of age (penicillin, cephalosporins and macrolides), excluding the attribution of this risk to metronidazole or quinolones. 

A limitation of this study is the lack of data on other possible maternal risk factors, such as breast feeding, maternal smoking habits during pregnancy, maternal body mass index and type of delivery, which could all be associated to IBD. 

A second limitation is the absence of data on antibiotic use in the entire population (available only for those born after 1995), thus reducing the sample size in the analyses. It was also not possible to determine whether antibiotics had been administered during hospitalizations. Due to the type of data codification, we were also are not able to distinguish between Crohn’s disease and ulcerative colitis among cases identified by means of healthcare co-payment exemptions.

Another limitation is the lack of information of the underlying infection that determined the use of antibiotics, that might be the actual responsible of the subsequent development IBD. We have adjusted for hospitalizations due to gastrointestinal infections, but most infections in children affect the upper respiratory tract and are often viral infections. In some cases, in fact, antibiotics could have been prescribed for non-bacterial infections. Finally, due to the clinical characteristics of the disease that mimic a gastroenteritis, it is not possible to exclude the presence of reverse causation and confounding. Immunological and inflammatory changes are probably present in IBD subjects prior to the development of the overt symptoms [44] and laboratory abnormalities may be found up to 12 months prior to IBD diagnosis [45]. However, we lack data from laboratory tests, such as faecal calprotectin, that could be useful to determine the amount of reverse causation. Therefore, antibiotics could have been prescribed for symptoms of undiagnosed IBD. Despite this may have occurred to some degree, due to the longitudinal study design, we believe the consistency of the risks observed with IBD onset even years after exposure, reinforced by the results of our sensitivity analyses, where the outcome occurred at least 1 year after exposure (onset between 2 to 5 years of age), suggest at most a marginal effect of these biases. 

## 5. Conclusions

Our study showed that perinatal characteristics were not associated to the onset of any type of any type of IBD. On the other hand, we have observed that exposure to elevated doses of antibiotics during the first year of life was associated with a higher risk of developing subsequent childhood onset IBD. The mechanisms by which antibiotics may predispose to IBD have not been clarified yet. However, evidence has shown that antibiotic use, especially in infancy, results in a change of the intestinal microbiome, and this may play a critical role on the development of the immune system. This is particularly relevant among children, potentially due to a more unstable nature of the infantile human microbiome. The dose-response association between antibiotics and IBD, particularly Crohn’s disease and EO-IBD, further suggest the importance of evaluating the benefits of antibiotic treatment, especially frequent prescriptions, among infants. 

## Figures and Tables

**Table 1:** Distribution of sex, year of birth and age at diagnosis IBD cases.

	All IBD Cases					IBD Cases for Antibiotic Exposure ^				
	Total (*n* = 164), (%)	EO-IBD † (n = 62), *n* (%)	P-IBD ‡ (*n* = 140), *n* (%)	Crohn’s Disease * (*n* = 83), *n* (%)	Ulcerative Colitis * (*n* = 63), *n* (%)	Total (*n* = 70), *n* (%)	EO-IBD † (*n* = 34), *n* (%)	P-IBD ‡ (*n* = 36), *n* (%)	Crohn’s Disease § (*n* = 38), *n* (%)	Ulcerative Colitis§ (*n* = 27), *n* (%)
Sex										
Male	95 (58)	38 (61)	57 (56)	52 (63)	34 (54)	44 (63)	23 (64)	21 (62)	26 (68)	16 (59)
Female	69 (42)	24 (39)	45 (44)	31 (37)	29 (46)	26 (37)	13 (36)	13 (38)	12 (32)	11 (41)
Calendar year of birth										
1989–1993	67 (41)	12 (19)	55 (54)	31 (38)	29 (46)	/	/	/	/	/
1994–1998	57 (35)	16 (26)	41 (40)	29 (35)	19 (30)	39 (56)	11 (31)	28 (82)	20 (53)	16 (59)
1999–2004	23 (14)	17 (27)	6 (6)	12 (14)	9 (14)	18 (26)	12 (33)	6 (18)	11 (29)	5 (19)
2005–2012	17 (10)	17 (27)	/	11 (13)	6 (10)	13 (19)	13 (36)	/	7 (18)	6 (22)

^ born after 1995. * among the 146 individuals with hospital admission diagnosis of Crohn’s disease or ulcerative colitis. § among the 65 individuals with hospital admission diagnosis of Crohn’s disease or ulcerative colitis, born after 1995. † Early Onset IBD: age at diagnosis <10 years. ‡ Pediatric IBD: age at diagnosis 10–17 years.

**Table 2:** Perinatal conditions and development of childhood onset IBD.

Characteristics	Cases (*n* = 164), *n* (%)	Controls (*n* = 1640), *n* (%)	Adj OR (95% CI)	Fully Adjusted OR (95% CI)
Season of birth				
May–September	76 (46.4)	700 (42.7)	1.16 (0.84–1.60)	1.19 (0.86–1.65)
October-April	88 (53.7)	940 (57.3)	1	1
Siblings				
Yes	66 (40.2)	762 (46.5)	1	1
No	98 (59.8)	878 (53.5)	1.29 (0.93–1.79)	1.21 (0.85–1.72)
Singleton/Multiple births				
1	159 (97.0)	1607 (98.0)	1	1
≥2	5 (3.1)	33 (2.0)	1.54 (0.59–4.03)	1.44 (0.49–4.18)
Birth weight				
≥2500	154 (93.9)	1563 (95.3)	1	1
<2500	10 (6.1)	77 (4.7)	1.31 (0.67–2.57)	1.12 (0.42–3.00)
Gestational age, weeks				
≥36	156 (95.7)	1586 (97.1)	1	1
≤35	7 (4.3)	47 (2.9)	1.50 (0.67–3.38)	1.26 (0.40–4.03)
Apgar score				
≥7	154 (93.9)	1542 (94.1)	1	1
<7	10 (6.1)	96 (5.9)	1.05 (0.53–2.07)	0.96 (0.47–1.96)
Maternal age, years				
<25	22 (13.4)	196 (12.0)	0.97 (0.58–1.62)	0.97 (0.57–1.63)
25–29	64 (39.0)	547 (33.4)	1	1
30–34	50 (30.5)	586 (35.7)	0.72 (0.49–1.07)	0.76 (0.51–1.14)
35–39	22 (113.4)	273 (16.7)	0.68 (0.41–1.14)	0.74 (0.43–1.27)
≥40	6 (3.7)	38 (2.3)	1.34 (80.54–3.31)	1.55 (0.61–3.91)
Maternal education				
Primary/middle school	62 (37.8)	673 (41.2)	1	1
High school	86 (52.4)	765 (46.8)	1.22 (0.86–1.72)	1.23 (0.87–1.76)
University	16 (9.8)	195 (12.0)	0.89 (0.50–1.58)	0.96 (0.53–1.76)

Adj: sex and year of birth. Fully adjusted: sex; year of birth; season of birth; having older siblings; number of births; birth weight; gestational age; Apgar scores at 1 minute; maternal age; and mother’s formal education at the moment of birth. OR: Odds ratio.

**Table 3:** Risks of antibiotic exposure in the first 6 and 12 months of life, in the development of childhood onset IBD.

	Cases (*n* = 70), *n* (%)	Controls (*n* = 700), *n* (%)	Adj OR (95% CI)	Adj 2 OR (95% CI)	Adj 3 OR (95% CI)
**Antibiotic prescription in the first 6 months of life**					
No	52 (74.3)	560 (80.0)	1	1	1
Yes	18 (25.7)	140 (20.0)	1.38 (0.79–2.43)	1.458 (0.81–2.63)	1.45 (0.80–2.62)
1 †	6 (8.6)	92 (13.1)	0.70 (0.29–1.67)	0.746 (0.31–1.81)	0.74 (0.31–1.81)
2–3 †	8 (11.4)	41 (5.9)	2.09 (0.95–4.64)	**2.29** (1.01–5.24)	2.31 (1.01–5.28)
≥4 †	4 (5.7)	7 (1.0)	**6.07** (1.76–20.96)	**6.25** (1.70–23.05)	**6.34** (1.68–24.02)
**Antibiotic prescription in the first 12 months of life**					
No	37 (52.9)	380 (54.3)	1	1	1
Yes	33 (47.1)	320 (45.7)	1.06 (0.65–1.73)	1.08 (0.64–1.80)	1.07 (0.64–1.79)
1 †	13 (18.6)	164 (23.4)	1.12 (0.57–2.18)	0.80 (0.40–1.58)	0.80 (0.40–1.58)
2–3 †	9 (12.9)	116 (16.6)	0.79 (0.37–1.70)	0.87 (0.40–1.88)	0.86 (0.40–1.87)
≥4 †	11 (15.7)	40 (5.7)	**3.75** (1.69–8.32)	**2.92** (1.32–6.46)	**2.91** (1.31–6.45)

† Reference group: not exposed to antibiotics; Adj: sex and year of birth Adj 2: sex; year of birth; season of birth; having older siblings; number of births; birth weight; gestational age; Apgar scores at 1 minute; maternal age; and mother’s formal education at the moment of birth. Adj 3: Adj 2 and gastrointestinal infections (hospital discharge record diagnosis).

**Table 4:** Risks of antibiotic exposure in the first 6 and 12 months of life, on the development of childhood onset IBD, stratified by EO-IBD and P-IBD.

	EO-IBD (1–9 Years)			P-IBD (10–17 Years)		
	Cases (*n* = 36) *n* (%)	Controls (*n* = 360) *n* (%)	Adj 2 OR (95% CI)	Cases (*n* = 34), *n* (%)	Controls (*n* = 340), *n* (%)	Adj 2 OR (95% CI)
**Antibiotic prescription in the first 6 months of life**						
No	24 (66.7)	292 (81.1)	1 (1–1)	28 (82.4)	268 (78.8)	1 (1–1)
Yes	12 (33.3)	68 (18.9)	**2.36** (1.06–5.24)	6 (17.7)	72 (21.2)	0.82 (0.31–2.13)
1 †	3 (8.3)	48 (13.3)	0.84 (0.24–2.99)	3 (8.8)	44 (12.9)	0.64 (0.18–2.26)
2–3 †	6 (16.7)	17 (4.7)	5.83 (1.77–19.18)	2 (5.9)	24 (7.1)	0.92 (0.21–4.12)
≥4 †	3 (8.3)	3 (0.8)	**15.07** (2.45–92.63)	1 (2.9)	4 (1.2)	2.43 (0.24–24.52)
**Antibiotic prescription in the first 12 months of life**						
No	17 (47.2)	201 (55.8)	1 (1–1)	20 (58.8)	179 (52.7)	1 (1–1)
Yes	36 (52.8)	159 (44.2)	1.43 (0.69–2.96)	14 (41.2)	161 (47.3)	0.81 (0.37–1.76)
1 †	6 (16.7)	84 (23.3)	0.86 (0.31–2.36)	7 (20.6)	80 (23.5)	0.74 (0.28–1.98)
2–3 †	3 (8.3)	60 (16.7)	0.64 (0.18–2.3)	6 (17.7)	56 (16.5)	1.14 (0.41–3.15)
≥4 †	10 (27.8)	15 (4.2)	**11.10** (3.41–36.13)	1 (2.9)	25 (7.4)	0.37 (0.05–2.97)

† Reference group: not exposed to antibiotics; Adj 2: sex; year of birth; season of birth; having older siblings; number of births; birth weight; gestational age; Apgar scores at 1 minute; maternal age; and mother’s formal education at the moment of birth.

**Table 5:** Risks of antibiotic exposure in the first 6 and 12 months of life, in the development of Crohn’s disease and ulcerative colitis.

	Crohn’s Disease (*n* = 38) *n* (%)	Controls (*n* = 380) *n* (%)	Adj 2 OR (95% CI)	UC (*n* = 27) *n* (%)	Controls (*n* = 270) *n* (%)	Adj 2 OR (95% CI)
**Antibiotic prescription in the first 6 months of life**						
No	25 (65.8)	313 (82.4)	1 (1–1)	22 (81.48)	207 (76.7)	1 (1–1)
Yes	13 (34.2)	67 (17.6)	**2.61** (1.17–5.81)	5 (18.52)	63 (23.3)	0.85 (0.29–2.52)
1 †	4 (10.5)	40 (10.5)	1.36 (0.42–4.34)	2 (7.41)	44 (16.3)	0.48 (0.10–2.26)
2–3 †	5 (13.2)	24 (6.3)	**3.30** (1.08–10.09)	3 (11.11)	15 (5.6)	2.40 (0.55–10.47)
≥4 †	4 (10.5)	3 (0.8)	**13.02** (2.47–68.47)	0 (0)	4 (1.5)	-
**Antibiotic prescription in the first 12 months of life**						
No	16 (42.1)	208 (54.7)	1 (1–1)	18 (66.67)	147 (54.4)	1 (1–1)
Yes	22 (57.9)	172 (45.7)	1.72 (0.84–3.53)	9 (33.33)	123 (45.6)	0.72 (0.28–1.86)
1 †	8 (21.1)	90 (23.7)	1.26 (0.50–3.19)	4 (14.81)	61 (22.6)	0.58 (0.17–1.97)
2–3 †	6 (15.8)	60 (15.8)	1.35 (0.49–3.70)	3 (11.11)	48 (17.8)	0.71 (0.18–2.78)
≥4 †	8 (21.1)	22 (5.8)	**4.86** (1.70–13.90)	2 (7.41)	14 (5.2)	1.42 (0.25–7.97)

† Reference group: not exposed to antibiotics. Adj 2: sex; year of birth; season of birth; having older siblings; number of births; birth weight; gestational age; Apgar scores at 1 minute; maternal age; and mother’s formal education at the moment of birth.

## Supplementary Materials

The following are available online at https://www.mdpi.com/1660-4601/17/7/2409/s1, Table S1: Perinatal risk factors and development of IBD in childhood, stratified by age at onset, Table S2: Risk of perinatal characteristics in the development of Crohn’s disease and UC, Table S3: Frequency of antibiotic prescriptions stratified by type of active ingredient and antibiotic spectrum, among IBD cases and controls, Table S4:. Risk of developing VEO-IBD (1-5 years) after antibiotic exposure in the first 6 and 12 months of life
