# Supplementary material for: Perinatal and Antibiotic Exposures and the Risk of Developing Childhood-Onset Inflammatory Bowel Disease: A Nested Case-Control Study Based on a Population-Based Birth Cohort
Source: Int J Environ Res Public Health. 2020 Apr 2;17(7):2409. doi: 10.3390/ijerph17072409 (PMC7177699; doi:10.3390/ijerph17072409)
Supplement: Supplementary file 1 [file ijerph-17-02409-s001.pdf]

Table S1 Perinatal risk factors and development of IBD in childhood, stratified by age at onset.

	**EO-IBD†**			**P-IBD‡**		
**Characteristics**	**Cases (n=62, n (%)**	**Controls (n=620), n (%)**	**Fully adjusted OR (95% CI)**	**Cases (n=102, n (%)**	**Controls (n=1020), n (%)**	**Fully adjusted OR (95% CI)**
**Season of birth**						
**May-September**	31 (50.0)	279 (45.0)	1.25 (0.74-2.12)	45 (44.1)	421 (41.3)	1.16 (0.77-1.76)
**October-April**	31(50.0)	341 (55.0)	1	57 (55.9)	599 (58.7)	1
**Siblings**						
**No**	36(58.1)	343 (55.3)	1.14 (0.65-1.99)	62 (60.8)	535 (52.4)	1.20 (0.76-1.90)
**Yes**	26 (41.9)	277 (46.7)	1	40 (39.2)	485 (47.6)	1
**Multiple births**						
**1**	61 (98.4)	610 (98.4)	1	98 (96.1)	997 (97.8)	1
**≥2**	1 (1.61)	10 (1.6)	0.89 (0.09-9.19)	4 (3.9)	23 (2.3)	1.68 (0.49-5.76)
**Birth weight**						
**≥2500**	58(93.6)	590 (95.2)	1	96 (94.1)	973 (95.4)	1
**<2500**	4 (6.5)	30 (4.8)	1.23 (0.31-4.96)	6 (5.9)	47 (4.6)	1.00 (0.25-4.05)
**Gestational age, weeks**						
**≤ 35**	2 (3.2)	12 (1.9)	1.35 (0.18-9.93)	5 (5.0)	35 (3.5)	1.41 (0.32-6.27)
**≥36**	60 (96.8)	606 (98.1)	1	96 (95.1)	980 (96.5)	1
**Apgar score**						

**≥7**	57 (91.9)	583 (94.0)	1	97 (95.1)	959 (94.2)	1
**≤ 6**	5 (8.1)	37 (6.0)	1.28 (0.46-3.50)	5 (4.9)	59 (5.8)	0.73 (0.27-2.00)
**Maternal age, years**						
**< 25**	5 (8.7)	54 (8.7)	0.86 (0.30-2.45)	17 (16.7)	142 (13.9)	1.00 (0.55-1.85)
**25-29**	19 (30.7)	178 (28.7)	1	45 (44.1)	369 (36.2)	1
**30-34**	23 (37.1)	245 (39.5)	0.99 (0.51-1.91)	27 (26.5)	341 (33.4)	0.68 (0.40- 1.14)
**35-39**	11 (17.7)	124 (20.0)	0.95 (0.42-2.14)	11 (10.8)	149 (14.6)	0.62 (0.30-1.28)
**≥ 40**	4 (6.5)	19 (3.1)	2.43 (0.70-8.36)	2 (2.0)	19 (1.9)	0.98 (0.22-4.46)
**Maternal education**						
**University**	6 (9.7)	105 (17.1)	0.63 (0.24-1.68)	10 (9.8)	91 (8.9)	1.40 (0.65-3.04)
**High school**	36 (58.1)	290 (47.1)	1.37 (0.77-2.44)	50 (49.3)	475 (46.7)	1.15 (0.74-1.81)
**Primary/middle school**	20 (35.3)	221 (35.9)	1	42 (41.8)	452 (44.4)	1

†Age at diagnosis <10 years

‡ Age at diagnosis 10-17 years

Fully adjusted: sex, year of birth, season of birth; having older siblings; number of births; birth weight; gestational age; Apgar scores at 1 minute; maternal age; and mother’s formal education at the moment of birth.

Table S2 Risk of perinatal characteristics in the development of Crohn's disease and UC.

	**Crohn's Disease**			**Ulcerative Colitis**		
**Characteristics**	**Cases (n=83, n (%)**	**Controls (n=830), n (%)**	**Adj2 OR (95% CI)**	**Cases (n=63, n (%)**	**Controls (n=630), n (%)**	**Fully adjusted OR (95% CI)**
**Season of birth**						
May-September	35 (42.17)	338 (40.7)	1.14 (0.71-1.82)	31 (49.21)	282 (44.8)	1.31 (0.77-2.22)
October-April	48 (57.83)	492 (59.3)	1	32 (50.79)	348 (55.2)	1
**Siblings**						
No	45 (54.22)	458 (55.2)	1.86 (0.52-1.40)	41 (65.08)	324 (51.4)	1.79 (0.99-3.22)
Yes	38 (45.78)	372 (44.8)	1	22 (34.92)	306 (48.6)	1
**Multiple births**						
1	79 (95.18)	818 (98.5)	1	79 (95.18)	612 (97.1)	1
≥2	4 (4.82)	12 (1.5)	2.18 (0.51-9.32)	1 (1.59)	18 (2.9)	1.58 (0.18-13.95)
**Birth weight**						
≥2500	74 (89.32)	795 (95.8)	1	63 (100.00)	595 (94.4)	1
<2500	9 (10.84)	35 (4.2)	2.01 (0.66-6.18)	0	35 (5.6)	-
**Gestational age**						
≤35	6 (7.23)	19 (2.3)	1.53 (0.37-6.42)	0	23 (3.7)	-
≥36	77 (92.77)	806 (97.7)	1	62 (100.00)	605 (96.3)	1

**Apgar**						
≥7	77 (92.77)	792 (95.4)	1	60 (95.24)	582 (92.7)	1
≤ 6	6 (7.23)	38 (4.6)	1.33 (0.52-3.38)	3 (4.76)	46 (7.3)	0.80 (0.22-2.86)
**Maternal age**						
< 25	11 (13.25)	95 (11.5)	1.08 (0.52-2.25)	7 (11.11)	79 (12.5)	0.66 (0.27-1-62)
25-29	33 (39.76)	281 (33.9)	1	25 (39.68)	203 (32.2)	1
30-34	19 (22.89)	297 (35.8)	0.48 (0.26-0.89)	24 (38.10)	221 (35.1)	1.07 (0.57-2.01)
35-39	15 (18.07)	133 (16.0)	0.80 (0.39-1.61)	6 (9.52)	114 (18.1)	0.62 (0.23-1.64)
≥ 40	5 (6.02)	24 (2.9)	1.46 (0.49-4.35)	1 (1.59)	13 (2.1)	1.05 (0.13-8.84)
**Maternal education**						
University	11 (13.25)	106 (12.8)	1.56 (0.70-3.52)	4 (6.35)	79 (12.5)	0.56 (0.18-1.70)
High school	46 (55.42)	396 (47.9)	1.61 (0.94-2.74)	34 (53.97)	282 (44.8)	1.22 (0.70-2.14)
Primary/middle school	26 (31.33)	324 (39.2)	1	25 (39.68)	269 (42.7)	1

Fully adjusted: sex, year of birth, season of birth; having older siblings; number of births; birth weight; gestational age; Apgar scores at 1 minute; maternal age; and mother's formal education at the moment of birth.

Table S3. Frequency of antibiotic prescriptions stratified by type of active ingredient and antibiotic spectrum, among IBD cases and controls.

ANTIBIOTIC ATC CODE (Active ingredient)	Antibiotic type	Spectrum (broad/narrow)	IBD			All per type of antibiotic
			Controls	Cases	All	
J01CA04 (amoxicillin)	penicillin	Broad	278	53	331	
			44.6	42.4		
J01CR02 (moxicillin and beta-lactamase inhibitor)		Narrow	56	12	68	
			9.0	9.6		399
J01DA06 (cefuroxime)	cephalosporin	Broad	5	0	5	
			0.8	0		
J01DA08-DC04 (cefaclor)		Broad	95	30	125	
			15.2	24.0		
J01DA11 (ceftazidime)		Broad	5	1	6	
			0.8	0.8		
J01DA23 (cefixime)		Broad	25	0	25	
			4.0	0		
J01DA33 (Cefpodoxime)		Broad	1	0	1	
			0.2	0		
J01DA39 (ceftibuten)		Broad	14	1	15	
			2.2	0.8		
J01DA41 (cefprozil)		Broad	1	0	1	
			0.2	0		
J01DC02 (cefuroxime)		Broad	1	0	1	
			0.2	0		
						193

**J01DD08 (cefixime)**		**Broad**	1	0	1	
			0.2	0		
**J01DD13 (cefpodoxime)**		**Broad**	2	1	3	
			0.3	0.8		
**J01DD14 (Ceftibuten)**		**Broad**	8	2	10	
			1.3	1.6		
**J01EA01 (trimethoprim)**	**sulfunamide**	**Broad**	1	0	1	
			0.2	0		
**J01EE01 (Sulfamethoxazole and trimethoprim)**		**Broad**	2	1	3	
			0.3	0.8		4
**J01FA01 (erythromycin)**	**macrolide**	**Narrow**	5	0	5	
			0.8	0		
**J01FA06 (roxithromycin)**		**Narrow**	1	0	1	
			0.2	0		
**J01FA07 (josamycin)**		**Broad**	17	1	18	
			2.7	0.8		
**J01FA09 (clarithromycin)**		**Narrow**	62	4	66	
			9.9	3.2		
**J01FA10 (azithromycin)**		**Narrow**	35	5	40	
			5.6	4		
**J01FA11 (miocamycin)**		**Narrow**	3	1	4	
			0.5	0.8		
**J01FA12 (rokitamycin)**		**Narrow**	3	0	3	
			0.5	0		137
**J01GB03 (gentamicin)**	**aminoglycoside**	**Broad**	0	10	10	
			0	8		
**J01GB07 (netilmicin)**		**Broad**	1	3	4	
			0.2	2.4		14
**J01MA01 (ofloxacin)**	**quinolone**	**Broad**	1	0	1	
			0.2	0		1

**J01XX01 (fosfomycin)**	**other**	**Broad**	1<br>0.2	0<br>0	1	1
**Total**			624	125	749	

Table S4 Risk of developing VEO-IBD (1-5 years) after antibiotic exposure in the first 6 and 12 months of life.

	**VEO-IBD (n = 19) n (%)**	**Controls (n = 190), n (%)**	**Adj 2 OR (95% CI)**
**Antibiotic prescription in the first 6 months of life**			
No	11 (57.9)	155 (81.6)	1
Yes	8 (42.1)	35 (18.4)	**4.56** (1.48-14.02)
1†	3 (15.8)	25 (13.2)	2.02 (0.49-8.40)
2-3†	3 (15.8)	9 (4.7)	9.32 (1.62-53.60)
≥4†	2 (10.5)	1 (0.5)	**25.63** (1.34-490.46)
**Antibiotic prescription in the first 12 months of life**			
No	5 (26.3)	108 (56.8)	1
Yes	14 (73.7)	82 (43.2)	**4.54** (1.47-14.02)
1†	5 (26.3)	45 (23.7)	3.42 (0.77-15.27)
2-3†	3 (15.8)	28 (14.7)	2.30 (0.47-11.18)
≥4†	6 (31.6)	9 (4.7)	**87.07 (8.43**-899.78)

†Reference group: not exposed to antibiotics

Adj2: sex, year of birth, season of birth; having older siblings; number of births; birth weight; gestational age; Apgar scores at 1 minute; maternal age; and mother's formal education at the moment of birth
